# Triple sexually transmitted infections among pregnant woman in the context of Elimination of mother to child transmission in Southern Ethiopia: Reports from a survey of questionnaires and laboratory studies.

**DOI:** 10.3389/fgwh.2023.1190170

**Published:** 2023-06-19

**Authors:** Eskinder Israel, Iskindir Hizkel, Temesgen Geta, Tihun Feleke, Beniyam Samuel, Desta Markos

**Affiliations:** ^1^ School of Public Health, College of Health Science and Medicine, Wolaita Sodo University, Wolaita Sodo, Ethiopia; ^2^ Department of Maternal and Child Health, Sawla Town Health Office, Gofa Zone, Sawla, Ethiopia; ^3^ School of Nursing, College of Health Science and Medicine, Wolaita Sodo University, Wolaita Sodo, Ethiopia; ^4^ Department of Nursing, Hawassa College of Health Sciences, Hawassa, Ethiopia; ^5^ Department of Midwifery, College of Medicine and Health Science, Dilla University, Dilla, Ethiopia

**Keywords:** Triple, STIs, Elimination, MTCT, pregnant woman, Southern Ethiopia

## Abstract

**Introduction:**

Sexually transmitted infections (STIs) cause a wide range of public health problems if left untreated. They can lead to adverse birth outcomes, including stillbirth, fetal loss, neonatal death, preterm birth, and low birth weight. Although great efforts have been made to reduce STIs nationally, their incidence remains high in Ethiopia, and their co-infection calls for urgent action. Therefore, this study aimed to identify the determinants of three STIs among pregnant women attending antenatal care (ANC) in the context of the elimination of mother-to-child transmission in public health facilities in Sawla Town, Gofa zone, Southern Ethiopia.

**Methods:**

A cross-sectional study design was conducted among pregnant women attending antenatal care in public health facilities in Sawla Town, Southern Ethiopia, from May to July 2022. Data were collected from pregnant women’s serum using an HIV rapid test, an HBsAg rapid test device, and a VDRL for HIV, HBV, and syphilis, respectively. Descriptive statistics, such as frequencies and percentages, were used to describe each relevant variable. Logistic regression analyses were used to identify the determinants of STIs.

**Results:**

A total of 484 pregnant women attending antenatal care were screened. The mean age of the women was 24.0 ± 4.6 years, and nearly half of the participants had completed secondary school or higher. The overall seroprevalence of three STIs (HIV, HBV, and syphilis) among pregnant women was 6.8%. These three sexually transmitted infections were shown to be more common among pregnant women who were not able to read and write, had tattoos, had previously had an abortion, and had a history of multiple sexual partners.

**Conclusions:**

The seroprevalence found in this study was intermediate in comparison with the WHO standard. Efforts should be made to strengthen the existing health education and RH service integration on STI screening, and treatment that further eliminates vertical infection.

## Quick points

The World Health Organization (WHO) has set a strategy and global target for the control and prevention of STIs, aiming for a 90% reduction in the incidence of syphilis and/or 50 or fewer cases of congenital syphilis per 100,000 live births in 80% of countries by 2030. If left untreated, STIs always result in a wide range of health problems.

## Introduction

More than one million sexually transmitted infections (STIs) are acquired every day worldwide (1, 2). A total of 340 million new cases of “curable” sexually transmitted infections (including syphilis) are expected to occur in men and women aged 15–49 years each year (2)**.** Sub-Saharan Africa, where most of this burden occurs, is at high risk of sexually transmitted infections (including human immunodeficiency virus (HIV), hepatitis B virus (HBV), and syphilis) (3). The majority of new STI cases in sub-Saharan African countries, including Ethiopia, occur among girls aged 15–19 years (3, 4).

Mother-to-child transmission (MTCT) of HIV remains the main route of HIV infection among children born to HIV-infected women and is a major public health problem in resource-limited settings (5). Viral hepatitis is the leading cause of human disease and is caused by different types of viruses, such as hepatitis A, B, C, D, E, and G. However, HBV, HCV, and HEV are known to co-infect with HIV (6–9). Hepatitis B virus infection in pregnancy is associated with a high risk of maternal complications and can cause adverse health outcomes ranging from acute hepatitis to chronic hepatitis, in addition to cirrhosis and hepatocellular carcinoma (HCC) (10). Syphilis in pregnancy can also result in adverse birth outcomes such as perinatal death, spontaneous abortion, and even neonatal death (11). Nowadays, co-infection with these three STIs in pregnancy has become a global concern for various reasons. First, HIV co-infection with HBV increases the MTCT of HBV compared to HBV transmission alone. Second, infants born to HBV- and syphilis-reactive women are at increased risk for MTCT of HIV compared to HIV alone (12, 13). The main modes of infection for these STIs are unsafe sexual contact, contaminated blood, and blood products, and vertical mother-to-child transmission (5, 14, 15). Without any intervention, MTCT is 15%–45% for HIV, 10%–90% for HBV, and 30%–100% for syphilis. However, it can be reduced to <5% (for breastfeeding) for HIV, 50 cases per 100,000 LB for syphilis, and 2% for HBV (16–18). STIs cause a wide range of public health problems if left untreated. They can lead to adverse birth outcomes, including stillbirth, fetal loss, neonatal death, preterm birth, and low birth weight (19).

A study undertaken from 2005 to 2014 to determine the trends and patterns of seroprevalence of three major STIs (HIV, hepatitis B virus (HBV), and syphilis) among pregnant women in Ethiopia showed a decreasing trend of >44% nationally: HIV (10.5%–5.5%), HBV (12.6%–6.7%), and syphilis (2.5%–1.1%) (20). This study also pointed out that the trend in HIV prevalence over time was higher in Gondar and Bahir Dar, while the lowest was recorded at the Health Center in Hawassa (20).

According to the 2011 Ethiopian Demographic and Health Survey (EDHS) report, the prevalence of HIV among the reproductive age group ranges from 0.9% in SNNPR to 5.2% in Addis Ababa and 6.5% in the Gambella region (21). The prevalence of hepatitis B among the reproductive age group is highest in SSA, where 5.4% were infected (22). Ethiopia, which also carries a great burden of this disease, was classified as a high HBV country in 2014 (23), with an overall rate of 6.7% in 10 major cities. Other recent evidence suggested that 7.8% of infants were stillborn or died within the first six weeks of life due to maternal syphilis (24). Despite regional differences in syphilis prevalence in sub-Saharan African countries (ranging from 2.1% in Eastern Africa to 2.4% in South Africa), the pooled prevalence reported was 2.87% (25). According to various studies, STIs are associated with physical, psychological, and social consequences that have a direct effect on the quality of life and sexual and reproductive health care (26).

To tackle this pandemic, the United Nations Development Programme (UNDP) has developed a number of Sustainable Development Goals (SDGs) to be achieved by 2030 (27). SDG 3 focuses on ending preventable deaths of children under five, combating communicable diseases, and providing universal access to sexual and reproductive health care (28). The World Health Organization (WHO) set a different strategy and global target for the control and prevention of STIs, such as a 90% reduction in syphilis incidence, and/or 50 or fewer cases of congenital syphilis per 100,000 live births in 80% of countries by 2030 (29). It also launched a global health initiative to further eliminate mother-to-child transmission of HIV, the hepatitis B virus, and syphilis through a triple elimination strategy (18). Based on the country’s previous experience and the benefit of scientific advances, the Ethiopian Federal Ministry of Health endorsed and committed to achieving the elimination of triple STIs (HIV, HBV, and syphilis) among pregnant women to reduce MTCT and ensure it is not a public health problem by 2025 (16). Despite ongoing efforts to lower STIs, their incidence, when combined with co-infections, remains high and calls for urgent action in Ethiopia. In line with this, studies have shown that the presence of triple STIs in pregnant women during pregnancy results in an increased risk of mother-to-child transmission (2, 18, 29). Therefore, this study aimed to identify determinants of triple STIs (HIV, HBV, and syphilis) among pregnant women attending antenatal care in public health facilities in Sawla Town, Gofa zone, Southern Ethiopia.

## Methods

### Study setting

Sawla is a new zonal town located in Gofa, Southern Ethiopia. It is located in Southwest Ethiopia, 515 km away from the center of the capital, Addis Ababa. The town has two health facilities, namely Sawla General Hospital (SGH) and Sawla Health Center. The mean expected ANC visit in Sawla Health Center (SHC) is 400 per month and 300 per month in Sawla General Hospital (SGH).

### Study design, period, and population

A cross-sectional study design was conducted from May to July 2022. All pregnant women who attended ANC at public health facilities in Sawla Town were the source population, while the study population consisted of all pregnant women who attended ANC at the time of data collection and met the inclusion criteria.

All pregnant women who attended ANC and gave written informed consent were included. Pregnant women who were in active labor had inconclusive test results and had lipemic, icteric, or hemolyzed samples that were excluded due to their interference with the test.

The sample size was initially calculated using the single population proportion formula,

$$\frac{n={\left(z_{\alpha /2}\right)}^{2}\times p(1-p),}{d^{2}}$$

where:

Z _1−_*_α_*_/2=_ significance level at *α* = 0.05 (standard normal variable at 95% confidence level = 1.96).

When computing with single population proportion formula, it yields small sample size (30–32). In order to get an adequate number of sample size, Epi-Info version was used. As a result, Epi Info version 7 was used to calculate the sample size for associated factors as follows (Table 1).

**Table 1:** Sample size calculation for an associated factor based on some variables from a review of studies using open Epi info version 7 for cross-sectional studies.

Factors	Percentage of unexposed with outcome	Two-sided confidence level	AOR	Power	Z*α*/2	Ratio of unexposed to exposed	Sample size	Ref. no.
History of punctures with sharp materials	1.5	95%	5.70	80	1.96	1	336	(55)
History of dental treatment	2.2	95%	4.10	80	1.96	1	404	(36)
History of abortions	1.6	95%	4.97	80	1.96	1	392	(56)
History of home delivery	2.01	95%	4.10	80	1.96	1	440	(36)

**Figure 1:**
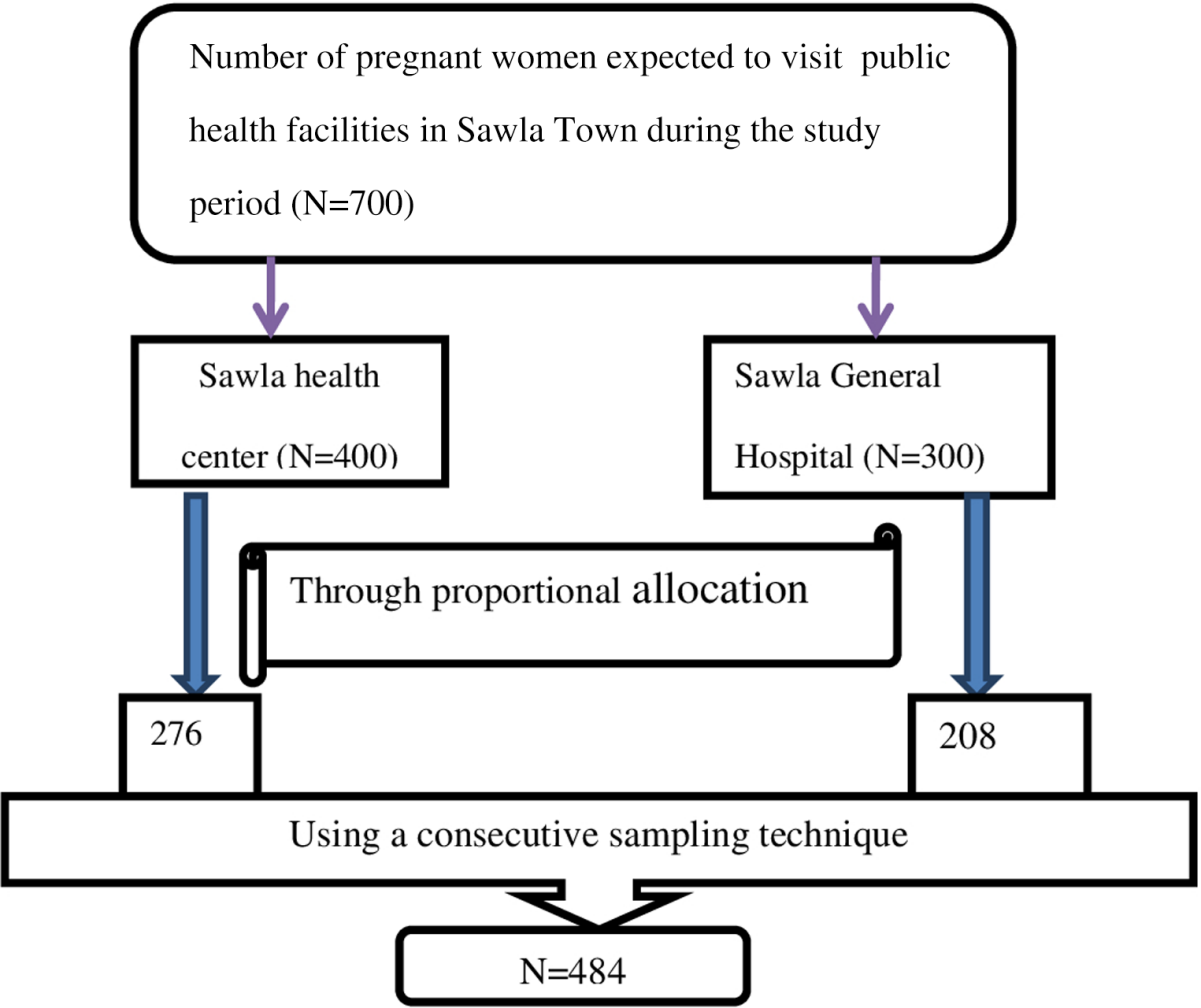
A figure describing sampling techniques and procedures used to select a total sample of 484 women attending antenatal care in public health facilities in Sawla Town, 2022.

The final sample size for this study was 484 after adding 10% of the non-response rate to the maximum sample size (440). It was also estimated from previous studies that 700 pregnant women visited Sawla Town’s public health facilities. The number of pregnant women along with their registration number was gathered from each health facility, and then a consecutive sampling technique was used to select study participants (Figure 1).

### Study variables/measures

Pregnant women’s STI-positive (HIV, HBV, syphilis) serostatus was taken as the dependent variable, while sociodemographic, behavioral, healthcare-related service, and obstetric and reproductive health characteristics were the independent variables in this study.

Data were collected using structured, pre-tested, and interviewer-administered questionnaires. These were initially developed by reviewing different related studies conducted on similar topics. It included sociodemographic characteristics (age, educational level, occupation, residence, marital status), behavior-related characteristics (multiple sexual partners, having ear piercings and tattoos, a habit of sharing sharp materials), healthcare-related characteristics (history of surgical procedures, history of dental care, history of catheterization, history of blood transfusion, history of hepatitis B vaccination), and obstetric and reproductive health-related characteristics (gravidity, parity, ANC visits, place of delivery, previous history of having an abortion, history of pregnancy-related complications). Data collectors and supervisors were trained by the principal investigator before data collection on how to ask and fill out the questionnaires and how to approach the women. The data collectors were Bsc midwives and Bsc medical laboratory technologists who had undergone basic STI training and worked in nearby hospitals (Arbaminch General Hospital) to minimize potential bias associated with the study.

### Laboratory testing

**Figure 2:**
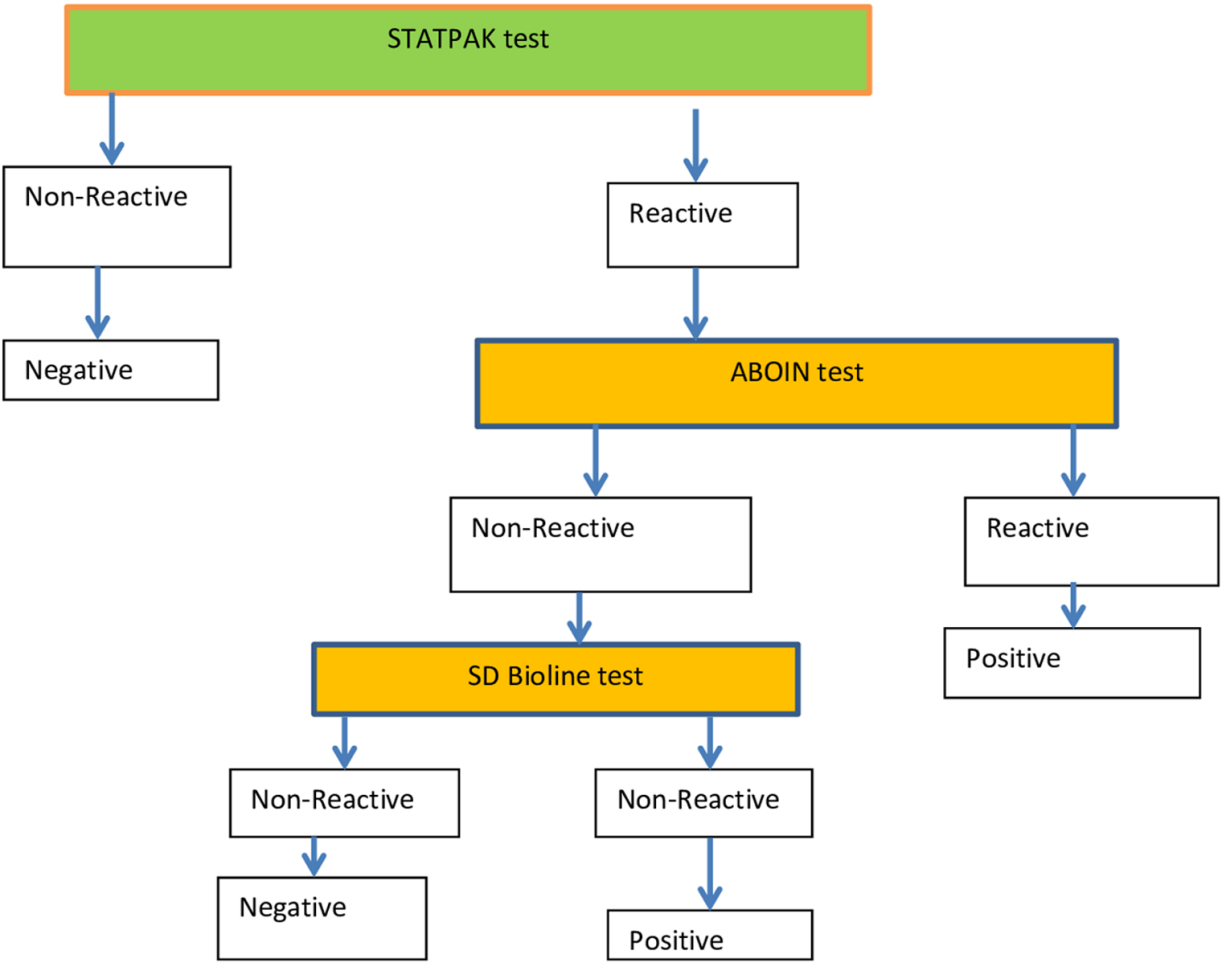
A flow chart showing the Current national testing algorithm for HIV.

Women who gave written informed consent were sent to the laboratory unit to provide a blood sample for serologic testing. At the laboratory unit, blood samples were collected aseptically from each pregnant woman in a properly labeled tube with the patient’s unique identification number (UIN). The samples were centrifuged and sera separated for processing, and positive samples were stored appropriately at an optimal temperature. The women were informed of the potential benefits and risks associated with the study before testing started to give the most accurate results possible. HIV testing was done using the current national HIV testing algorithm (Figure 2). Testing for HBV in pregnant women was performed with the use of the HBsAg Rapid Test Device. Serum samples from pregnant women for syphilis testing were collected using a Venereal Disease Research Laboratory (VDRL) test strip, and in this case, too, interpretation of the results was performed based on the manufacturer’s instructions.

#### Hepatitis B virus (HBV) testing

The sera from pregnant women’s samples were screened using the HBsAg Rapid Test Device according to the manufacturer’s instructions and laboratory procedures. All standard operating procedures were followed step-by-step. To ensure the quality of the procedure, three negative and two positive controls were run simultaneously with the test procedures.

#### Syphilis testing

The sera from pregnant woman samples were analyzed using a Syphilis Rapid Test Strip (One Step Strip Style ANTI-TP) to detect antibodies produced against Trepollema palladium, and interpretation of results was performed based on the manufacturer’s instructions.

### Quality assurance and quality control in the laboratory

Standard operating procedures were strictly followed in the laboratory unit. To ensure the controlled performance of our testing procedure, we primarily used in-house controls for both negative and positive control samples in addition to controls provided by the manufacturers. As a result, reagents and test methods were assessed with these known positive and negative control materials to evaluate the storage conditions of reagents and the performance capability of the method. Positive samples were confirmed by specific test procedures and analyzed separately. Finally, the results were checked by supervisors.

## Operational definitions

• Seroprevalence of HBV, HIV, and syphilis: Pregnant women’s combined prevalence of at least one of three STIs (Hepatitis B, HIV, or syphilis) for a serologic test (33).
• Seropositive for HIV: Status of pregnant women that tested positive for HIV (33, 34).
• Seropositive for hepatitis B virus: status of pregnant women who tested positive for HBsAg (34).
• Seropositive for syphilis: status of pregnant women who tested positive for the syphilis rapid test (33, 34).

### Statistical approach

First, the collected data were checked for completeness. Then, these were coded, entered into Epidata 7.2.2.6, and exported to the SPSS version 25 statistical package for further analysis. Descriptive statistics, such as frequencies and percentages, were computed. Bivariate logistic regression analysis was performed first, and variables with a *p*-value <0.25 were considered candidates for multivariate logistic regression. Finally, those variables that were significant with a *p*-value <0.05 were considered determinants of STIs.

### Ethical clearance

Ethical clearance was initially obtained from the Institutional Review Board (IRB) of Hawassa University, College of Medicine and Health Science, with Ref. No. IRB/154/13. Letters of support were also obtained from the Gofa Zone Health Department and Sawla Town Health Office to undertake a study prior to data collection. The purpose of the study was explained to each pregnant woman, and they were informed about anonymity and their right not to respond to specific questions if they felt uncomfortable. Before and during data collection, women were asked for both permission and written informed consent. Informed consent was provided by legally authorized representatives for women aged 15–18 years. To ensure the privacy and confidentiality of client information, a unique identification number and a separate room were used during the interview. All procedures were carried out in accordance with relevant guidelines and regulations.

## Results

### Women’s sociodemographic characteristics

Data from 484 pregnant women were assessed in this analysis. A total of 469 (97%) pregnant women had complete data. Of these, 444 (94.7%) were married, with the majority of them aged between 15 and 24 years, with a mean (±SD) age of 24.04 ± 4.6 years. Education-wise, 191 (40.7%) participants completed their secondary education or higher. In total, 192 (40.9%) subjects were housewives, with the majority (85.1%) residing in urban areas (Table 2).

**Table 2:** Socio-demographic characteristics of pregnant women attending ANC at public health facilities in Sawla town, Gofa zone, southern Ethiopia, 2022.

Variable	Category	Frequency	Percentage
Age	15–24	232	49.5
	25–34	227	48.4
	>35	10	2.1
Education	Not able to read and write	43	9.2
	Able to read and write	42	9
	Primary	130	27.7
	Secondary	191	40.7
	College and above	63	13.4
Occupation	Employee	221	47.1
	Housewife	192	40.9
	Student	56	11.9
Residence	Urban	399	85.1
	Rural	70	14.9
Marital status	Married	444	94.7
	Unmarried	12	2.6
	Divorced	09	1.9
	Informal union	04	0.9

### Behavioral characteristics of women

**Figure 3:**
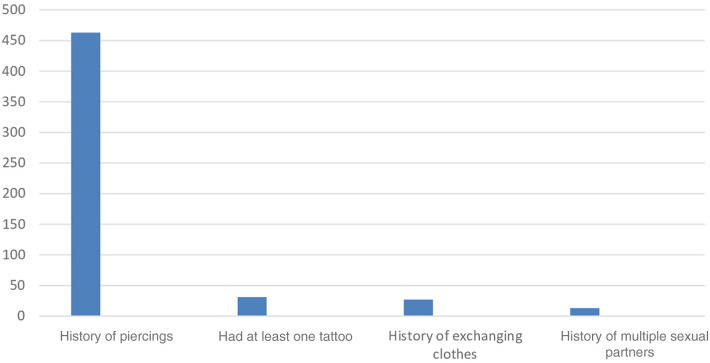
A figure showing behavioral risk factors for pregnant women attending antenatal care at public health facilities in Sawla Town, Gofa zone, Southern Ethiopia, 2022.

Of the total 469 pregnant women who attended ANC, 463 (98.3%) had their ear lobes pierced, and 31 (6.6%) had at least one tattoo (Figure 3).

### Health care related information about the women

In total, 457 (97.4%) women reported that they had no previous history of surgical procedures. Conversely, 42 (9%) subjects had a history of dental treatment, while 21 (4.5%) had a history of catheterization. More than 5% (*n* = 444) of pregnant women in this study had a history of blood transfusion. Regarding hepatitis vaccination, 13 (2.8%) pregnant women were vaccinated, while 61 (13%) had a previous history of STIs (Table 3).

**Table 3:** Healthcare services for pregnant women attending ANC at public health facilities in Sawla town, Gofa zone, southern Ethiopia, 2022.

Variable	Category	Frequency	Percentage
History of surgical procedures	Yes	12	2.6
	No	457	974
Dental care	Yes	42	9
	No	427	91
Catheterization	Yes	21	4.5
	No	448	955
History of blood transfusions	Yes	25	5.3
	No	444	947
Hepatitis B vaccination	Yes	13	2.8
	No	456	972
History of STIs	No	408	87
	Yes	61	13

### Obstetric and reproductive health characteristics of women

A total of 198 (42.2%) pregnant women were gravida III and 197 (42%) were para II during this index pregnancy. In total, 142 (30.3%) participants attended their ANC at least once during this study. Almost all (93.4%) pregnant women gave birth in health facilities. In total, 49 (10.4%) pregnant women had a history of unsafe abortion, and 26 (5.5%) had pregnancy-related complications (Table 4).

**Table 4:** Obstetric and reproductive characteristics of pregnant women attending ANC at public health facilities in Sawla town, Gofa zone, southern Ethiopia, 2022.

Variable	Category	Frequency	Percentage
Gravidity	I	95	20.3
	II	151	32.2
	III	198	42.2
	IV and above	25	5.3
Parity	0	103	22
	I	146	31.1
	II	197	42
	III and above	23	4.9
ANC visits	1st visit	142	30.3
	2nd visit	107	22.8
	3rd visit	166	35.4
	4th visit and above	53	11.3
Place of delivery	Home	31	6.6
	Health facility	438	93.4
History of abortions	Yes	49	10.4
	No	420	89.6
History of pregnancy-related complications	Yes	26	5.5
	No	443	94.5

### Overall seroprevalence of HIV, HBV, and syphilis

**Figure 4:**
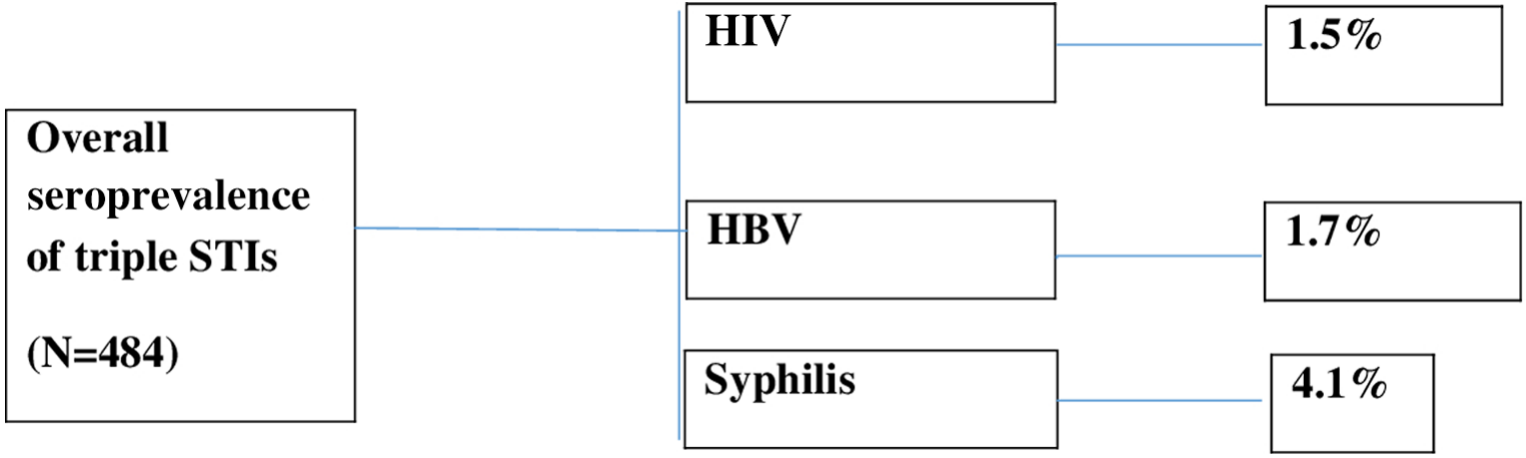
A flow chart to show the seropositive prevalence of STIs from the study.

A total of seven (1.5%) pregnant women were found to be HIV positive, eight (1.7%) were HBV positive, and 19 (4.1%) were found to be reactive to the syphilis test at the end of the study. The overall seroprevalence of triple STIs (HIV, HBV, and syphilis) among pregnant women in this study was 6.8% (Figure 4).

### Determinants of STIs (HIV, HBV, and syphilis) in pregnant women

In the bivariate analysis, four variables with a *p*-value < 0.25 were selected as candidate variables for multivariable logistic regression analysis. These were: maternal educational status; tattoos; history of abortions; and history of multiple sexual partners.

After controlling for the effect of confounding variables in multivariable logistic regression analysis, pregnant women who were not able to read and write were four times more likely to have an STI compared to their counterparts (AOR = 4.1 CI = 2.3–24.3) *P* = 0.001. Pregnant women who had tattoos had a 5-fold increased chance of acquiring an STI than their counterparts (AOR = 5.2 CI = 1.8–11.3) *P* = 0.001. The odds of women who had a previous history of having an abortion increased the likelihood of getting an STI by two times more than their counterparts (AOR = 2.7 CI = 1.3–5.5) *P* = 0.001. Compared to pregnant women who had no history of multiple sexual partners, having a history of multiple sexual partners increased the odds of acquiring an STI 5-fold (AOR = 5.2 CI = 2.1–14.4) *P* = 0.01 (Table 5).

**Table 5:** Determinants of triple STIs (HIV, HBV, and syphilis) among pregnant women attending ANC in public health facilities in Sawla town, southern Ethiopia, 2022.

Variable	Category	STI status		COR(95% CI)	AOR (95% CI)
		Positive	Negative		
		(%)	(%)		
Educational level	Not able to read and write	06 (1.3%)	37 (7.9%)	6.2 (3.4–32.1)*	4.1 (2.3–24.3)**
	Able to read and write	01 (0.2%)	41 (8.7%)	1.5 (0.1–24.8)	1.3 (0.8–19.1)
	Qmentary	08 (1.7%)	122 (26%)	4.1 (0.5–33.2)	3.8 (0.4–29.2)
	Secondary	16 (3.4%)	175 (37.3%)	5.7 (0.7–43.6)	5.4 (0.5–41.1)
	Tertiary	01 (0.2%)	62 (13.2%)	1	1
Tattoos	Yes	09 (1.9%)	22 (4.7%)	7.3 (3.1–17.8)*	5.2 (1.8–11.3)**
	No	23 (4.9%)	415 (88.5%)	1	1
History of abortions	Yes	08 (1.7%)	41 (8.7%)	3.2 (1.4–7.6)*	2.7 (1.3–5.5)**
	No	24 (5.1%)	396 (84.4%)	1	1
History of multiple sexual partners	Yes	06 (1.3%)	13 (2.8%)	7.5 (2.6–21.4)*	5.2 (2.1–14.4)**
	No	26 (5.5%)	424 (90.4%)	1	1

* Variables statistically significant with *p*-value < 0.25.

** Variables statistically significant with *p*-value < 0.05.

## Discussion

This study aimed to examine the determinants of triple STIs (HIV, HBV, and syphilis) among pregnant women attending antenatal care in public health facilities in Sawla Town, Gofa zone, southern Ethiopia.

In this study, the overall seroprevalence of triple STIs (HIV, HBV, and syphilis) among pregnant women attending antenatal care was 6.8%. This shows that encouraging progress has been made in this area in recent years toward the global goal of eliminating mother-to-child transmission by 2030. This finding is higher than studies conducted in India (4.8%) and Iran (1.2%) (19, 35). This discrepancy may be due to differences in sample size, study area, and study population. India and Iran used the general population to calculate the proportion, whereas our study was only limited to pregnant women attending care in public health facilities. The seroprevalence of HIV found in this study was 1.5%. This indicates the need to strengthen the existing health education package on HIV testing, modes of transmission, and prevention of mother-to-child transmission of HIV in the area to further reduce infection**.** This prevalence is lower than what was observed in studies from Gondar Referral Hospital (10.3%), Gondar (11.2%), Dessie (6.5%), and Abuja, Nigeria (11.5%) (33, 36–38). This may be due to differences in access to healthcare facilities, health-seeking behaviors, and socio-cultural practices such as early sexual initiation leading to increased sexual intercourse and infection with STIs. This seroprevalence may also be influenced by the small sample size. This is also the case for other studies conducted in the country.

The seroprevalence of HBV among pregnant women in the current study was 1.7%. Despite these data, HBV testing coverage is very low in the Ethiopian context, which shows that considerable work should be done on increasing the capability and efficiency of both health workers and healthcare facilities to meet the global target of eliminating HBV by 2030. This study finding is lower when compared with findings from Gambela Hospital, Adigrat General Hospital, Arba Minch Hospital, Dawuro zone, Nigeria, Kenya, Tanzania, and Ghana, which reported prevalences of 7.9%, 9.2%, 4.3%, 3.5%, 3.9%, 3.8%, 3.9%, and 7.7%, respectively (24, 31, 39–44). This observed variation may be due to differences in socio-demographic, educational, sociocultural, and behavioral factors. The current study showed that 4.1% of pregnant women were reactive to syphilis. This is lower than what was observed in studies from Yirgalem Hospital (5.1%) and Cameroon (5.7%) (32, 45). However, it is higher than the data collected during studies conducted in Debre Berhan (1.8%) (34), Bahir Dar (2.9%) (46), Dessie (0.6%) (37), Felege Hiwot Referral Hospital (2.6%) (46), and Eastern Africa (2.2%) (25). These observed differences may be the result of varying access to STI treatment, risky sexual behavior, sociocultural variations, and the use of a small sample size. The Ethiopian Ministry of Health’s STI guidelines recommend that testing for syphilis be done in antenatal care as a routine practice. Separately, recent data conducted in Ethiopia in 2019/2020 revealed that only two-thirds of pregnant women were screened for syphilis (47). Therefore, this suggests the need to ensure that all pregnant women receive syphilis screening and be treated for syphilis if found positive.

In the current study, pregnant women who were not able to read and write were four times more likely to get an STI compared to their counterparts. This is similar to the study conducted in the Tertiary Health Institution of Benin City, Nigeria, and the Netherlands (41, 48). This is because illiterate pregnant women are less aware of their health problems and less likely to seek medical attention at an early stage, which increases their risk of contracting STIs.

Pregnant women who had tattoos had a 5-fold increased risk of getting STIs compared to their counterparts. This is similar to studies of systematic reviews and meta-analyses and available studies (49–51). This could be due to the introduction or piercing of different instruments if these are reused without proper disinfection procedures.

Women with a history of abortions were twice as likely to get an STI as their counterparts. This finding is in line with the study conducted in Dawuro, Ethiopia, and the Shandong province, China (24, 52, 53). The scientific explanation for this could be that having a history of abortions makes women very susceptible to sexually transmitted infections. When an unsafe abortion does occur, it may be performed by unskilled and/or traditional practitioners, without adherence to aseptic techniques, further facilitating the transmission of STIs (2, 26).

Additionally, a history of multiple sexual partners increases the probability of acquiring an STI fivefold compared to pregnant women who have no history of multiple sexual partners. This finding is supported by evidence from southwest Nigeria, Gondar, and Tanzania (41, 54). This may be because women with multiple sexual partners are less likely to use condoms when compared with others and are at greater risk for STIs (2, 29).

This study tried to identify determinants of Human Immunodeficiency Virus, Hepatitis B Virus, and syphilis transmission among pregnant women attending antenatal care in both hospitals and health centers. However, it has several limitations. First, since it is a cross-sectional study, a strong causal association cannot be inferred. Second, as the information was collected only from pregnant women who had access to health facilities at the time of data collection, some women were left out, resulting in an underestimation of the results. Third, the study did not specify a time of STI serotransmission among women that occurred during pregnancy, and/or delivery, and/or the postpartum period. Finally, the study may have been subject to recall and social desirability bias because of the sensitive nature of some of the questions. Despite these limitations, the study sought key reasons, such as what causes triple STIs (HIV, HBV, and syphilis) among pregnant women attending antenatal care in the study area. The determinants found in this study can best be addressed through careful planning and implementation at all levels of health care.

## Conclusions

The seroprevalence found in this study was intermediate when compared to the WHO standard. Efforts should be made to strengthen the existing health education and RH service integration on STI screening and treatment to further eliminate vertical transmission. Triple sexually transmitted infections were shown to be more common among pregnant women who were not able to read and write, had tattoos, had previously had an abortion, and had a history of multiple sexual partners.

## Data Availability

The raw data supporting the conclusions of this article will be made available by the authors, without undue reservation.
